# The effect of O-antigen length determinant *wzz* on the immunogenicity of *Salmonella* Typhimurium for *Escherichia coli* O2 O-polysaccharides delivery

**DOI:** 10.1186/s13567-023-01142-4

**Published:** 2023-02-27

**Authors:** Yue Han, Ping Luo, Huan Zeng, Pu Wang, Jiali Xu, Pengju Chen, Xindan Chen, Yuji Chen, Qiyu Cao, Ruidong Zhai, Jing Xia, Simin Deng, Anchun Cheng, Changyong Cheng, Houhui Song

**Affiliations:** 1 Key Laboratory of Applied Technology On Green-Eco-Healthy Animal Husbandry of Zhejiang Province, Zhejiang Provincial Engineering Research Center for Animal Health Diagnostics & Advanced Technology, Zhejiang International Science and Technology Cooperation Base for Veterinary Medicine and Health Management, China-Australia Joint Laboratory for Animal, Health Big Data Analytics, College of Animal Science and Technology & College of Veterinary Medicine, Zhejiang A & F University, 666 Wusu Street, Hangzhou, 311300 China; 2 Institute of Preventive Veterinary Medicine, College of Veterinary Medicine, Sichuan Agricultural University, Chengdu, 611130 China; 3 Henan Institute of Morden Chinese Veterinary Medicine, Zhengzhou, 450002 China; 4 Shangdong Xindehui Biotechnology Co., Ltd, Yunchengxian, 274700 China

**Keywords:** *Salmonella* Typhimurium, lipopolysaccharide, O-antigen chain length, *Escherichia coli* O2, immune response

## Abstract

Attenuated *Salmonella* Typhimurium is a promising antigen delivery system for live vaccines such as polysaccharides. The length of polysaccharides is a well-known key factor in modulating the immune response induced by glycoconjugates. However, the relationship between the length of Lipopolysaccharide (LPS) O-antigen (OAg) and the immunogenicity of *S*. Typhimurium remains unclear. In this study, we assessed the effect of OAg length determined by *wzz*_ST_ on *Salmonella* colonization, cell membrane permeability, antimicrobial activity, and immunogenicity by comparing the *S*. Typhimurium wild-type ATCC14028 strain to those with various OAg lengths of the Δ*wzz*_ST_ mutant and Δ*wzz*_ST_::*wzz*_ECO2_. The analysis of the OAg length distribution revealed that, except for the very long OAg, the short OAg length of 2–7 repeat units (RUs) was obtained from the Δ*wzz*_ST_ mutant, the intermediate OAg length of 13–21 RUs was gained from Δ*wzz*_ST_::*wzz*_ECO2_, and the long OAg length of over 20 RUs was gained from the wild-type. In addition, we found that the OAg length affected *Salmonella* colonization, cell permeability, and antibiotic resistance. Immunization of mice revealed that shortening the OAg length by altering *wzz*_ST_ had an effect on serum bactericidal ability, complement deposition, and humoral immune response. *S*. Typhimurium mutant strain Δ*wzz*_ST_::*wzz*_ECO2_ possessed good immunogenicity and was the optimum option for delivering *E. coli* O2 O-polysaccharides. Furthermore, the attenuated strain ATCC14028 Δ*asd*Δ*crp*Δ*cya*Δ*rfbP*Δ*wzz*_ST_::*wzz*_ECO2_-delivered *E. coli* O2 OAg gene cluster outperforms the ATCC14028 Δ*asd*Δ*crp*Δ*cya*Δ*rfbP* in terms of IgG eliciting, cytokine expression, and immune protection in chickens. This study sheds light on the role of OAg length in *Salmonella* characteristics, which may have a potential application in optimizing the efficacy of delivered polysaccharide vaccines.

**Supplementary Information:**

The online version contains supplementary material available at 10.1186/s13567-023-01142-4.

## Introduction

Antimicrobial resistance represents an enormous global health crisis and one of the most serious public health issues today [1]. *Salmonella* outbreaks linked to backyard poultry in the United States increased during the COVID-19 pandemic in 2020 [2]. Avian pathogenic *Escherichia coli* (APEC) O2, a predominant serogroup of extraintestinal pathogenic *E. coli*, causes severe systemic infectious diseases in poultry because multidrug-resistant *E. coli* can live in chicken respiratory microbiota, which forms competitive exclusion of normal bacteria [3]. As a result, immunization against salmonellosis and colibacillosis is still required in poultry farms.

Because the outer leaflet of the outer membrane is primarily made up of LPS, surface polysaccharides are promising targets for vaccine development [4]. LPS typically consists of three components: lipid A, the core oligosaccharide, and O-antigen. The OAg moiety of LPS has been identified as a significant target for protective immunity against several pathogens based on many biological features of LPS OAg, including the following: (a) it is required for the physiological integrity and functionality of most Gram-negative bacterial outer membranes, which serve as a permeability barrier and contribute significantly to structural integrity; (b) it is a primary surface antigen, one of the most effective stimulators of the host immune system and plays a critical role in bacterial pathogen-host interactions; and (c) antibody specific to the OAg confers pathogen protection and contributes to complement and bactericidal peptide resistance. Throughout the last few decades, some studies have focused on changing the structure of LPS as a technique for developing live-attenuated vaccines [5].

OAg length is a well-known factor that influences the immunological response by altering the immunogenicity of the outer membrane antigen. Thus, changing the OAg length has previously been used to overcome T-independent immune responses [6]. However, the impact of OAg length on polysaccharide vaccine immunogenicity is still being debated. In some cases, low molecular-mass OAg fragments induce significantly higher antibody levels in mice than full-length OAg. For example, *Shigella sonnei* conjugates with low molecular-mass OAg fragments producing significantly higher antibody levels in mice than full-length OAg (29 RUs) [7]. A synthetic sequence of OAg with 3 RUs from *Shigella flexneri* serotype 2a conjugated to tetanus toxoid (TT) increases immunogenicity and protective efficacy in mice. While the anti-LPS antibody titers in mice and rabbits increase with OAg length in *S*. Typhimurium. A glycoconjugate with native OAg provides better protection than a smaller conjugate [8]. Furthermore, according to a recent study, OAg length is not a critical parameter for generalized modules for membrane antigen (GMMA) immunological response [9]. Although OAg length undoubtedly impacts the immunogenicity of polysaccharide (PS) vaccines, the optimal length of attenuated *Salmonella* vector for oral delivery of OAg has yet to be determined.

The Wzz family of polysaccharide co-polymerases (PCP) acts as a major hub in the OAg elongation process, regulating the degree of repeat unit polymerization (i.e., OAg size) [10]. Wzz proteins confer a wide range of model lengths, from 4 to > 100 RUs [11]. For example, there are three OAg lengths in *Salmonella*: short (S-OAg, < 15 RUs), long (L-OAg, 16 to 35 RUs), and very long (VL-OAg, > 100 RUs), with *wzz*_ST_ controlling the lower modal length pattern and *fepE* controlling the high molecular weight (HMW) LPS modal length (> 100 RUs) [12]. In *E. coli*, however, OAg length was classified as short (7–16 RUs), intermediate (10–18 RUs), and long (16–25 RUs) [13]. In a previous study, we discovered that the OAg lengths of wild-type *E. coli* O2 and *S*. Typhimurium differed in silver-stained SDS-PAGE [14]. When O2 OAg was synthesized in *S*. Typhimurium, the OAg length was controlled by *S*. Typhimurium Wzz_ST_, resulting in an OAg length that differed from that of wild-type *E. coli* O2. Although it is involved in *S*. Typhimurium pathogenesis, the role of *fepE* in immune responses is still unclear [15, 16]. Thus, we would like to alter *wzz*_ST_ to explore the effect of the OAg length on the immunogenicity of both the *Salmonell*a vector and the delivered OAg in the presence of *fepE*.

This study aimed to investigate how different OAg lengths affect *S*. Typhimurium immunogenicity and host defense resistance, as well as a promising application in heterologous polysaccharide delivery.

## Materials and methods

### Bacterial strains, plasmids, and growth conditions

The strains and plasmids used in this study are listed in Table 1. Bacterial strains were grown in Luria–Bertani broth or on agar plates (Sangon Biotech (Shanghai) Co., Ltd) at 37 °C. Diaminopimelic acid (DAP) (50 µg/mL) (Sigma-Aldrich) was added to the Δ*asd* mutant strains for growth in the absence of plasmid complementation. In the allelic exchange experiments, LB agar containing 5% sucrose was used for *sacB* gene-based counterselection [17]. Chloramphenicol (25 µg/mL) (Sangon Biotech (Shanghai) Co., Ltd) was used to select mutant strains for construction.

**Table 1:**
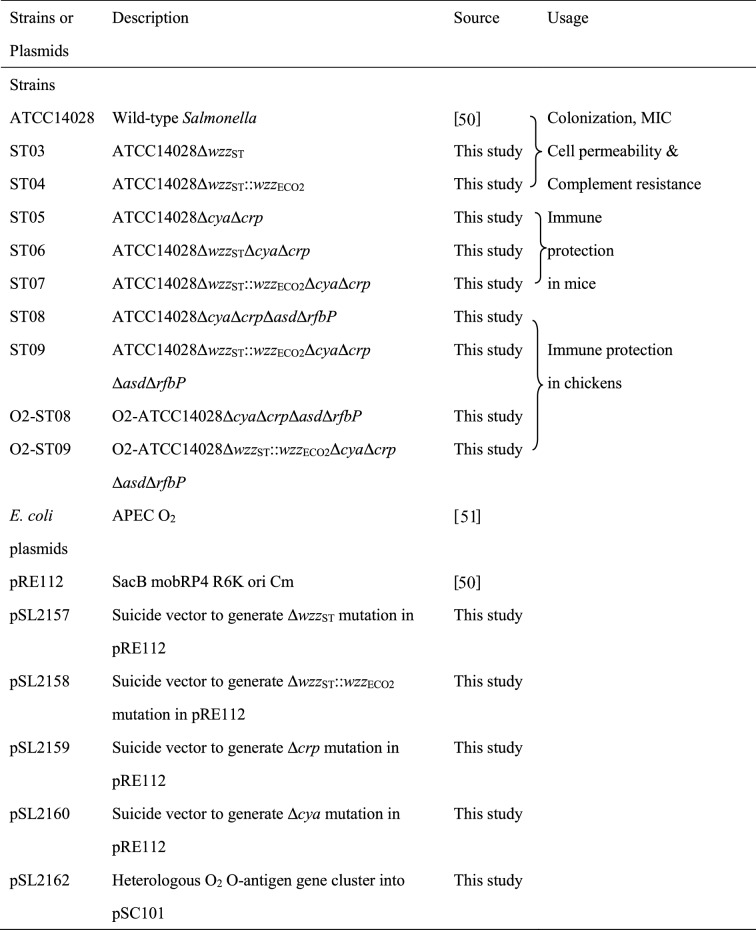
**Bacterial strains and plasmids used in this study**

### Generation of suicide vectors and mutant strains

The primers used in this study are listed in Table 2. DNA manipulations were carried out as described previously [18]. The *wzz*, *crp*, *cya*, *rfbP*, and *asd* genes were disrupted in *S*. Typhimurium by allelic exchange with a SacB-based suicide plasmid. The conjugation was then accomplished through an allelic exchange, as previously reported. For an insertion mutation, the Δ*wzz*_ST_ mutant strain was then inserted with heterologous *wzz* genes from *E. coli* O2. The CDS sequence of *wzz*_ECO2_ was fused with the upstream and downstream of the *S*. Typhimurium *wzz*_ST_ using primer pair *wzz*_ECO2_-F/*wzz*_ECO2_-R, which was designed with a homologous sequence with an adjacent fragment and plasmid pRE112. The same methods as described for the deletion mutant were applied to successfully select the Δ*wzz*_ST_::*wzz*_ECO2_ substitution mutation, including colony PCR, sequencing, and silver staining.

**Table 2:** **Primers used in this study**

Primers	Sequence (5′–3′)	Target Gene/host gene	GenBank nnununumbernu number
*wzz-1F*	TGGCTCCGATAACTTCCGCG	Upstream of *wzz*_ST_	NC_003197.22
*wzz-1R*	AGATACCCTAACTAAAAAAAG		
*wzz-2F*	GCTGCTTTGGCGACGCCAGC	Downstream of *wzz*_ST_	NC_003197.22
*wzz-2R*	GCTTCTTTGCCGGATGGTGG		
*wzz*_*ECO2*_*-F*	CATCCTTTTTTTAGTTAGGGTATCTATGCGGACTTGGAAATTTCC	DE17 *wzz*	CP045206.1
*wzz*_*ECO2*_*-R*	ACCATCCGGCAAAGAAGC TTACTTCGCGTTGTAATTGCG		
Ch-*β-actin-F*	CACCACAGCCGAGAGAGAAAT	Chicken	L08165.1
Ch-*β-actin-R*	TGACCATCAGGGAGTTCATAGC		
Ch-IL2-F	GCTAATGACTACAGCTTATGGAGCA	Chicken	GU119890.1
Ch-IL2-R	TGGGTCTCAGTTGGTGTGTAGAG		
Ch-IL4-F	GCGTCAAGATGAACGTGACAGA	Chicken	CP100567.1
Ch-IL4-R	GGAGCTGACGCATGTTGAGG		
Ch-IL10-F	CATGCTGCTGGGCCTGAA	Chicken	LR633956.1
Ch-IL10-R	CGTCTCCTGATCTGCTTGATG		
Ch-IFN-γ-F	CTGGAATCTCATGTCGTTCATCG	Chicken	NM_205149.2
Ch-IFN-γ-R	AGTCGTTCATCGGGAGCTTG		

*Ch* chicken.

### LPS profile analysis

The effect of heterologous *wzz* on OAg length was confirmed using silver-stained SDS-PAGE and Western blotting. LPS samples were prepared, separated, and visualized in the same manner as previously described [19]. The extracted LPS samples were transferred to PVDF membranes and incubated with *Salmonella* O4 or antiserum against *E. coli* O2 (Tianjin Biochip Corporation, Tianjin, China) at a 1:1000 dilution to identify the biosynthesis of *S*. Typhimurium and *E. coli* O2 OAg chains.

### Complement resistance assay

The serum complement resistance of ATCC14028, ST03, and ST04 with varying *wzz* was investigated as previously described [13, 20]. In brief, mid-log phase bacteria were washed twice with phosphate-buffered saline (PBS) buffer (pH 7.0), and 50 μL of the bacterial suspension (10^4^ CFU/mL) was mixed with an equal volume of 20% normal rabbit serum (NRS) (Cedarlane) (test group) or heat-inactivated rabbit serum (HIRS) (negative control) and incubated at 37 ℃ for 60 min. The same bacteria incubated in PBS was set as a blank control. The colony-forming units (CFU) produced by each treatment were determined by serial dilutions plated on LB agar medium and incubated at 37 °C overnight till the colony was viable to count. The strains had been tested three times. The survival rates were calculated by comparing the colony numbers that survived in the test group to those in the negative control group. The statistical difference between the wild-type strain and the *wzz*_ST_ mutant strains was compared by t-test using the Graphpad Prism.

### MIC and antibiotic sensitivity testing

The minimum inhibitory concentrations (MIC) of polymyxin B and bile salt deoxycholate (DOC) (Sigma-Aldrich) against ATCC14028, ST03, and ST04 were determined as previously described [21]. Along with the 96-well plates, two-fold serial dilutions of DOC (0.39–50 mg/mL) and polymyxin B (0.078–10 µg/mL) were made. Bacteria were grown in LB to an *OD*_600_ of 0.8, harvested, washed twice with PBS, and diluted in PBS to 1.0 × 10^5^ CFU/mL. Then, 100 μL of diluted bacteria suspension was added to each well containing an equal amount of antimicrobial substance at a different concentration, and overnight incubation at 37 ℃ was performed. As a negative control, groups with a single polymyxin B/DOC or bacteria were used. The optical density of each culture was determined using a SYNERGY^H1^ Microplate Reader (BioTek Instrument, Winooski, VT, USA). The threshold of inhibition was 0.1 at *OD*_600_. Actual titers were determined by spreading culture dilutions onto LB plates followed by overnight incubation at 37 °C. This test was repeated three times.

Furthermore, antibiotic sensitivity to Sulfamethoxazole (STX), Ampicillin (Amp), Chloramphenicol (Cm), Norfloxacin (NOR), Penicillin (P), and Tetracycline (TE) was detected with antibiotic discs (Hangzhou Microbial Regent Co., Ltd).

### Colonization in mice

Six-week-old female BALB/c mice were purchased from Hangzhou Medical College (Zhejiang, China). There are three groups of mice used to detect colonization. Each group of six mice was orally inoculated with 20 μL of (buffered saline with gelatin) BSG, which contained 1 × 10^9^ CFU of ATCC14028, ST03, and ST04, respectively. On days 6 and 9 post-infection, Peyer patches, spleen, and liver samples were collected from three mice of each group. To determine the viable bacteria, samples were homogenized, diluted, and plated onto XTL4, MacConkey, and LB agar.

### Cell membrane permeability

Bacteria were cultured overnight in LB broth without shaking. 1 mL of culture was diluted in 19 mL of fresh LB broth and cultivated at 37 °C with stirring until the optical density at 600 nm reached 1.8. 10 mL of cells were harvested by centrifugation at room temperature and washed twice with 50 mM potassium phosphate buffer (pH 7.0) at room temperature. After 20-fold dilution, the cells were resuspended in 0.5 mL of the same buffer, and the optical density was determined. Cells with a total of 0.4 *OD*_600_ units were added to the same potassium phosphate buffer (final volume, 2 mL). After the addition of ethidium bromide at a final concentration of 6 μM to the mixture. The fluorescence of the ethidium-nucleic acid complex generated by the influx of ethidium into cells was measured at room temperature using a spectrofluorometer with excitation and emission wavelengths of 545 and 600 nm, respectively [22]. The value was read every 70 s for 30 min, and the test was repeated three times. ATCC14028 cells were suspended in a 50 mM potassium phosphate buffer without Ethidium bromide as a blank control. The positive control was ATCC14028 cells treated with 75% ethanol for 1 h.

### Immunoprotection evaluation in mice

To determine the effect of the *wzz* mutant strains on *Salmonella* immunogenicity, the *wzz*_ST_^+^ and *wzz*_ST_ mutant strains were attenuated by deleting the *crp* and *cya* genes from the genome, as the Δ*cya*Δ*crp* mutation strain was a promising *Salmonella* vector for oral delivery of vaccine antigen [23]. The LPS profile of each attenuated strain was analyzed by silver staining and Western blotting.

Female ICR mice (4–6-week-old) were obtained from Hangzhou Medical College (Zhejiang, China). The ICR mice were randomly divided into four groups (10 per group), and each group was inoculated orally with 20 μL of BSG containing approximately 1 × 10^9^ CFU of ST05, ST06, ST07, and BSG only, respectively. The second immunization was carried out 14 days later with the same dosage. Blood samples of five mice in each group were collected from retro-orbital puncturing seven days after the booster immunization. Then, each group was challenged orally with 5 × 10^7^ CFU of ATCC14028 (~ 100 times LD_50_) 14 days after the booster injection.

As described previously, serum antibody concentrations were determined using a quantitative enzyme-linked immunosorbent assay (ELISA) [14]. *S*. Typhimurium LPS was extracted and coated on microtiter plates at a concentration of 1 µg/mL as previously described [24]. The plates were incubated overnight at 4 °C before being blocked for 2 h at room temperature with PBS containing 5% bovine serum albumin (BSA). Then, for the LPS-coated wells, 100 μL of a 1:100 diluted serum was applied in triplicate to individual wells. Goat anti-mouse IgG, IgG1, IgG2a, and IgA (Abcam, Shanghai, China) were sequentially added to the wells, followed by a *p*-nitrophenyl phosphate substrate (Sigma-Aldrich, St. Louis, MO, USA). Color development was recorded at 405 nm using a SYNERGY^H1^ microplate reader (BioTek Instrument, Winooski, VT, USA).

### Serum bactericidal activity (SBA) and complement deposition detection

The serum samples obtained from mice were inactivated at 56 °C for 30 min, and mid-log phase bacteria were collected for tenfold serial dilution. Sera samples were two-fold serially diluted from 1:25 to 1:100. Then, 10 µL of diluted bacteria (10^4^ CFU/mL) were mixed with 50 µL inactivated serum, 25 µL of rabbit complement (Cedarlane), and 15 µL of PBS and incubated for 1 h at 37 °C. The positive control was a mixture of bacteria and rabbit complement with inactivated rabbit anti-serum against *Salmonella* O4 (Tianjin Biochip Corporation, Tianjin, China). The blank control was bacteria in PBS buffer, and the negative control was a mixture of bacteria and complement. Then, the mixture was serially ten-fold diluted and spread on LB agar plates. Bacterial counts and survival rates were calculated by comparing the viable count to the blank control, and statistical analysis was performed by the t-test using Graphpad Prism.

The complement deposition ability was detected using inactivated sera. 1 mL of mid-log phase bacterial culture was washed and resuspended in PBS. After that, 50 µL of the bacterial sample was centrifuged and resuspended in 100 µL of 10% complement-inactivated sera for a 30 min incubation at 37 °C. The samples were then washed, resuspended, and incubated for 30 min at 37 °C in PBS containing 30 µL of rabbit complement. After another PBS wash, the samples were stained for 30 min in the dark on ice with 100 µL of FITC-conjugated goat anti-rabbit complement C3 (1:200 dilution, MP Biomedicals), resuspended in 2% formaldehyde, and analyzed by flow cytometry (BD FACSVerse™). The negative control was wild-type *S*. Typhimurium incubated with non-vaccinated complement-inactivated mouse sera.

### Cellular stimulation and cytokine detection in mouse splenic CD4^+^ T cells

The preparation of single-cell spleens was carried out as previously described [25]. 7 days after the boost immunization, splenocytes were stimulated with *S*. Typhimurium LPS (50 ng/µL) for 8 h at 37 °C with 5% CO_2_ in the presence of a protein transport inhibitor mixture (Thermo Fisher). Cytokine expression of CD4^+^ T cells was measured using rat anti-mouse CD3-Alexa Fluor 700, CD4-FITC, IFNγ-APC/Cy7, and IL-4-Alexa Fluor 647 (eBioscience). The ratio of cytokine-positive cells in the unstimulated sample was subtracted from the antigen-stimulated value of the same mouse to calculate the percentage of antigen-specific cells.

### The biosynthesis of *E. coli* O2 OAg in attenuated *Salmonella* and chicken immune protection

To determine whether attenuated *Salmonella* ST09 (ATCC14028 Δ*wzz*_ST_::*wzz*_*ECO2*_Δ*asd*Δ*crp*Δ*cya*Δ*rfbP*), with a medium OAg length (13–21 RUs), was a better choice for *E. coli* O2 delivery than ST08 (ATCC14028 Δ*asd*Δ*crp*Δ*cya*Δ*rfbP*), with a long OAg length (native RUs), *E. coli* O2 OAg was biosynthesized in attenuated *S*. Typhimurium ST08 and ST09, and the immune protection assay in chickens was performed.

One-day-old Xiaoshan chickens were purchased from Hangzhou Xiaoshan Donghai culture Co. Ltd. After the first seven days of free-range raising, the chickens were divided randomly into four groups. The regimens were prime immunized at 7 days of age and boosted with 1 × 10^8^ CFU/100 µL oral gavage at 14 days of age. Ten days after the second immunization, serum was collected from five chickens in each group. The remaining chickens were challenged with 1 × 10^9^ CFU of the APEC O2 strain via injection, and mortality was recorded daily for 2 weeks. Immune responses were measured in the same manner as previously described [26]. Immune sera were collected from the jugular veins and diluted in steps ranging from 1:100 to 1:800. The LPS-specific antibody was detected with goat anti-chicken IgY (Abcam, Shanghai, China). The ELISA titer refers to the highest dilution of sera with an OD_405_ value exceeding 2.1-fold above the negative control. Furthermore, chickens were weighed once a week during the feeding period.

### mRNA level of classic cytokines in chicken spleen

Four genes of IL-2, IL-4, IL-10, and IFN-γ were chosen for qPCR analysis to determine the mRNA levels of those involved in the immune response. Spleens were taken 7 days after the APEC O2 challenge. Total RNA was dissolved in Tris–EDTA (pH 7.5) buffer after extraction with the FastPure Cell/Tissue Total RNA Isolation Kit (Vazyme). The concentration of RNA was determined using the SYNERGY^H1^ Microplate Reader (BioTek Instrument). cDNA was generated from 1 µg of total RNA using Hiscript III reverse transcriptase (Vazyme). The Hiscript III All-in-one RT SuperMix Perfect for qPCR (Vazyme) was used to examine the mRNA transcripts of IL-2, IL-4, IL-10 and IFN-γ, as well as the housekeeping β-actin. Target gene transcripts were normalized to the housekeeping gene β-actin. The relative expression was calculated using the comparative method of 2^−ΔΔCt^, where ΔCt = Ct (target gene)-Ct (housekeeping gene), ΔΔCt = ΔCt (test sample)- ΔCt (reference sample).

### Histopathological and immunohistochemical staining of the liver and spleen from chickens

Surviving chickens from each group were chosen randomly for histopathological and immunohistochemical staining 7 days after the APEC O2 wild-type strain DE17 challenge. The liver and spleen were collected and fixed in 4% buffered formalin for 24–48 h before being stained with hematoxylin and eosin (HE). Tissue samples from the BSG-immunized group served as negative controls.

Immunohistochemistry was performed on formalin-fixed and paraffin-embedded tissue sections (4 μm). Sections of liver and spleen tissues were immunostained to evaluate the expression and distribution of the *E. coli* O2 antigen. The primary antibody was rabbit polyclonal against *E. coli* O2 (Tianjin Biochip Corporation, Tianjin, China) (1:100 dilution), and the secondary antibody was HRP-conjugated goat anti-rabbit (Servicebio) (1:200 dilution). The immunoreactivity was then visualized using 3,3-diaminobenzidine tetra-hydrochloride (DAB). The nucleus was counterstained with hematoxylin and photographed with Leica Microsystems at 200 × and 400 × magnification. Image-pro plus 6.0 was used for image analysis.

### Statistical analysis

Data were analyzed using GraphPad Prism 5 software (Graph Software, San Diego, CA, USA) and expressed as the means ± SD. A two-sample t-test was used for comparison between two groups, and ANOVA was used for multi-group comparison. *P* < 0.05 was considered statistically significant.

## Results

### Construction and identification of mutant strains

**Figure 1:**
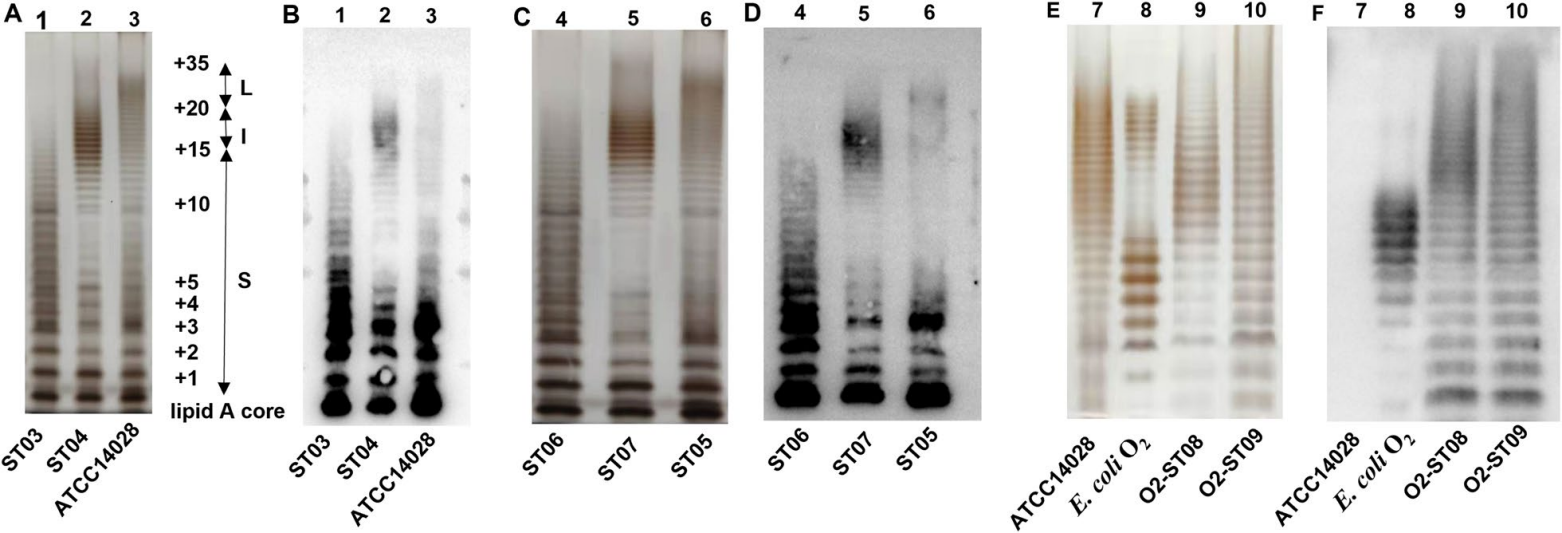
**Analysis of LPS profiles from *S***. **Typhimurium with various OAg lengths**. **A** Identification of gene replacement mutants by silver-stained SDS-PAGE. The lower band was unsubstituted lipid A-core, whereas the upper cluster of bands showed LPS with different lengths of OAg. Lane 1, 2 and 3 represents ST03 (Δ*wzz*_ST_), ST04 (Δ*wzz*_ST_::*wzz*_ECO2_) and ATCC14028, respectively. ST03 (Δ*wzz*_ST_) was assigned into short OAg length group (< 15 RUs), ST04(Δ*wzz*_ST_::*wzz*_ECO2_) was assigned into intermediate OAg length group (15–20 RUs), and the RUs of WT strain ATCC14028 was assigned into long OAg length group (20–35 RUs). **B** Western blotting detection against anti-*Salmonella* O_4_ serum. **C** LPS profiles of attenuated *S*. Typhimurium with different OAg lengths. **D** Identification of panel C by Western blotting. Lane 4, 5, 6 represents ST06 (ATCC14028Δ*wzz*_ST_Δ*crp*Δ*cya*), ST07 (ATCC14028Δ*wzz*_ST_::*wzz*_ECO2_Δ*crp*Δ*cya*) and ST05 (ATCC14028Δ*crp*Δ*cya*), respectively. **E** LPS profiles of *E. coli* O2 synthesized in *S*. Typhimurium ST08 (ATCC14028Δ*wzz*_ST_Δ*crp*Δ*cya*Δ*asd*Δ*rfbP*) and ST09 (ATCC14028Δ*wzz*_ST_::*wzz*_ECO2_Δ*crp*Δ*cya*Δ*asd*Δ*rfbP*). Lane 7, 8, 9 represents ATCC14028, *E. coli* O2, O2-ST08, and O2-ST09, respectively. **F** Western blotting of LPS with rabbit antiserum against serotype O2 *E. coli*.

In order to obtain *S*. Typhimurium with various OAg lengths, the *wzz*_ST_ was first deleted from ATCC14028 by homologous recombination, yielding the Δ*wzz*_ST_ mutant strain namely ST03, then, the *wzz*_ECO2_ was amplified by primer *wzz*_ECO2_-F/R, cloned, and inserted into the pRE112 suicide plasmid. After conjugative transfer, organisms containing heterologous *wzz*_ECO2_ were screened and sequenced. If the inserted sequence was identical to *wzz* from *E. coli* O2, the genetic substitution mutant Δ*wzz*_ST_::*wzz*_ECO2_ was successfully constructed and named ST04. Silver-stained SDS-PAGE (Figure 1A) and Western blotting (Figure 1B) were used to further examine the LPS profile of each strain. The LPS OAg length was categorized according to the OAg length classification method both in *Salmonella* and *E. coli* [13]. ST03 (lane 1) showed an LPS ladder with 2–7 RUs, which was assigned to the short OAg length group (< 15 RUs). ST04 (lane 2), showing an LPS ladder with 13–21 RUs, was assigned to the intermediate OAg length group (15–20 RUs). Wild-type ATCC14028 (lane 3) had a similar LPS ladder with 16–35 RUs, and was assigned to the long OAg length group (20–35 RUs).

The above strains of ST03, ST04, and ATCC14028 were further attenuated by deleting *crp* and *cya* from the genome, generating ATCC14028Δ*wzz*_ST_Δ*cya*Δ*crp*, ATCC14028 Δ*wzz*_ST_::*wzz*_ECO2_Δ*cya*Δ*crp*, and ATCC14028Δ*cya*Δ*crp*. After PCR determination and Sanger sequencing, the attenuated strains were named ST06, ST07 and ST05, respectively. The LPS profile of the attenuated strains was further confirmed by silver staining (Figure 1C) and Western blotting (Figure 1D). The profile shows that bacterial OAg length was not affected after attenuation (Figures 1A and C). The result reveals that the OAg length is independent of *crp* and *cya* attenuation.

To deliver *E. coli* O2 OAg polysaccharide, the balanced-lethal host-vector system based on *asd*, which encodes diaminopimelic acid (DAP), was further constructed, ensuring the stability of the heterologous plasmid pSL2162 in bacterial hosts without antibiotic pressure. The *rfbP* gene encodes UDP-phosphate galactose phosphotransferase, which is responsible for the transfer of galactose to the Undpp, *rfbP* deletion results in the absence of OAg in *Salmonella* LPS. Thus, the *asd* and *rfbP* genes were further deleted from the strains ST05 and ST07, resulting in ST08 (ATCC14028Δ*cya*Δ*crp*Δ*asd*Δ*rfbP*) and ST09 (ATCC14028 Δ*wzz*_ST_::*wzz*_ECO2_Δ*cya*Δ*crp*Δ*asd*Δ*rfbP*).

Then the *E. coli* O2 OAg carried by pSL2162 was electrotransformed into ST08 and ST09, yielding recombinant strains O2-ST08 and O2-ST09. The LPS profiles of O2-ST08 and O2-ST09 with different OAg lengths (lanes 9 and 10) were also detected by silver staining and Western blotting (Figures 1E, F). According to the results, the length of heterologously expressed *E. coli* O2 OAg length is determined by the Wzz. The O2-ST09 had 13–21 RUs, whereas the O2-ST08 had 16–35 RUs, which were used to evaluate the effect of OAg length on the protective efficacy of *E. coli* O2 in chickens.

### The effect of OAg length on *S*. Typhimurium colonization in mice, cell membrane permeability, antibiotic resistance and complement resistance

**Figure 2:**
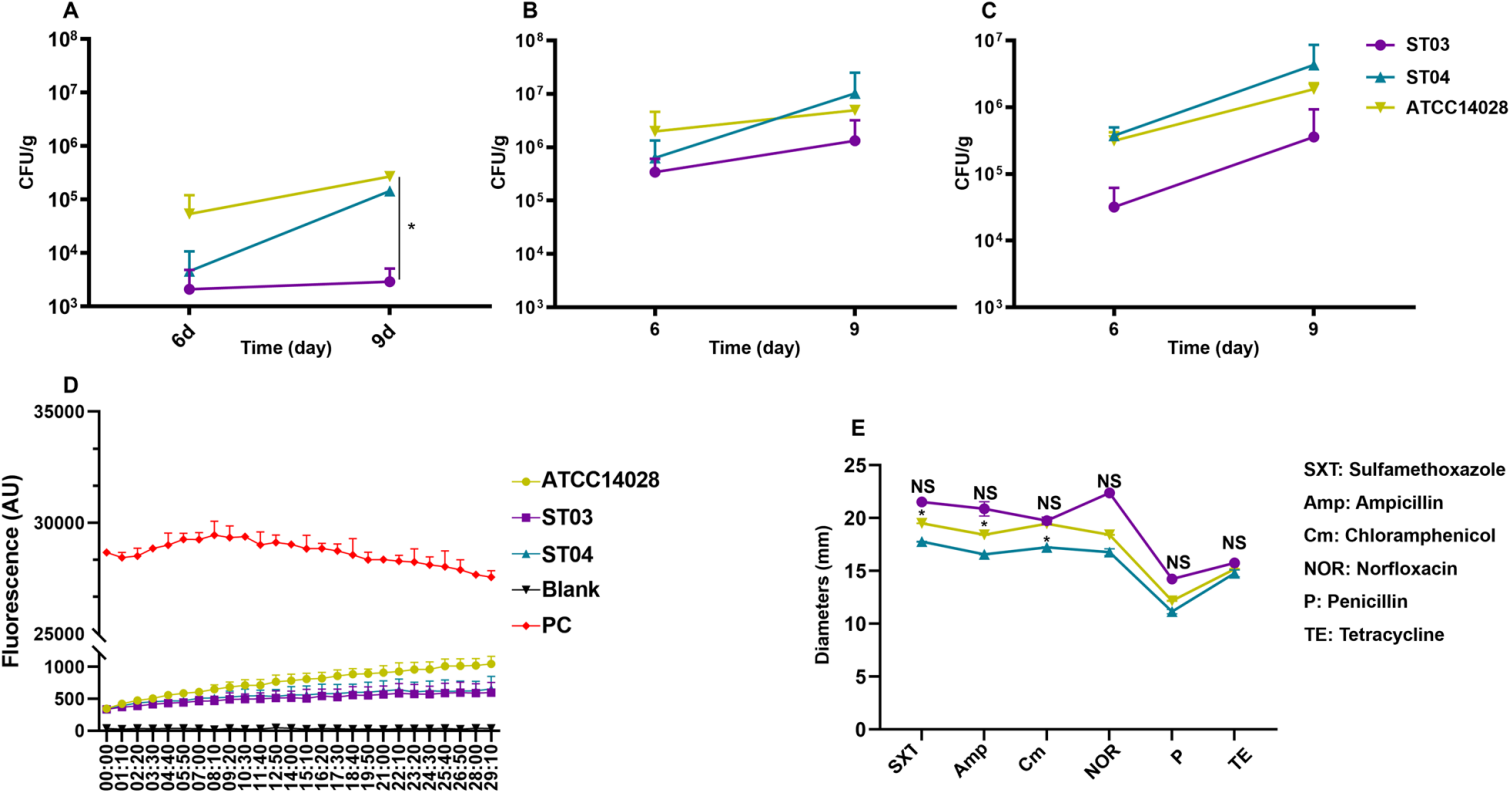
**The impacts of OAg length on bacterial colonization in mice, cell permeability and antibiotic resistance.** Colonization of ATCC14028, ST03 and ST04 in mice intestines (**A**), spleens (**B**), and livers (**C**). **D** Membrane permeability of ATCC14028, ST03 and ST04. The study was performed by recording the fluorescence of Ethidium influx into *wzz* substitution mutant strains at 600 nm with excitation at 545 nm. Blank control is ATCC14028 suspended in 50 mM potassium phosphate buffer without Ethidium bromide, PC control is ATCC14028 treated with 75% ethanol for 1 h. **E** Antibiotic sensitivity assay. The inhibitory zone diameter for each strain was measured. Error bars represent the standard deviation of 3 replicate samples.

LPS is an essential virulence factor that promotes infection in a mouse model. Therefore, we evaluated the effects of OAg length on bacterial colonization ability in BALB/c mice and discovered that the total bacterial load increased from 6 to 9 days post-infection (dpi). For colonization in intestinal cells (Figure 2A), no statistical difference was observed at 6 dpi, while the ST04 group shows a lower colonization than the WT strain ATCC14028 (*P* < 0.05) and a higher level than ST03 at 9 dpi (*P* < 0.01). The ST03 group was attenuated by more than one order of magnitude compared with that at 9 dpi. Thus, the bacterial colonization ability in the intestine in order from high to low is ATCC14028, ST04 and ST03. The same trend was also observed in the spleen (Figure 2B) and liver (Figure 2C) colonization, except for the data at 9 dpi, which shows that ST04 exceeded ATCC14028, as two mice died in the WT group due to high bacterial load (LD_50_ = 5 × 10^5^ CFU). Overall, the results show that OAg length influenced bacterial colonization ability in mice.

LPS, the major component of outer membranes, also acts as a permeability barrier. To investigate the effect of OAg length on *S*. Typhimurium membrane permeability, ethidium bromide was used as a fluorescent probe for outer membrane (OM) bilayer permeability. The membrane permeability tests show that the blank control of ATCC14028 without ethidium bromide had the lowest fluorescence value and the PC control (ethanol-treated strain) had the highest fluorescence value, indicating that the test results were valid. The fluorescence value of the three tested strains, from highest to lowest, were ATCC14028, ST04 and ST03. The fluorescence value of ATCC14028 was more than twice as high as that of ST03 (Figure 2D), indicating that OAg length does impact cell membrane permeability [27].

As cell permeability affects antibiotic resistance, we also measured antibiotic sensitivity to SXT, Amp, Cm, NOR, P, and TE (Figure 2E). The diameters of the tested strains for SXT, from high to low, were ST03, ST04 and ATCC14028. ST04 exhibits greater resistance to SXT than ATCC14028 (*P* < 0.05). The same trend was also seen in the Amp, Cm, NOR, P and TE sensitivity detection. Although all three detected strains met the criteria for identifying antibiotic sensitivity and resistance, the data show that OAg length modification affected bacterial antibiotic resistance.

In addition, the MIC for DOC (an anionic surfactant) and polymyxin B (a cationic antimicrobial peptide) were also measured. We discovered that the MIC value of wild-type ATCC14028 to polymyxin B was 1.25 µg/mL, while ST03 and ST04 show a MIC value of 0.625 µg/mL, making them more sensitive to polymyxin B than wild-type ATCC14028, suggesting that the *wzz*_ST_ gene contributed to the sensitivity to polymyxin B. Regarding the DOC sensitivity, ST04 has a MIC value of 50 mg/mL, higher than that of ATCC14028 and ST03, with a MIC value of 25 µg/mL (Table 3).

**Table 3:** **MIC of DOC and Polymyxin B, swimming motility of wild type *Salmonella*** **and its derivatives**

Strain	O-antigen length (RUs)^a^	MIC		Complement resistance (%)^c^
		DOC (mg/mL)^b^	Polymyxin B (μg/mL)	
Δ*rfbP*	0	6.25	0.625	9.54 ± 2.4
ST03	2–7	25	0.625	58.48 ± 8.9
ST04	13–21	50	0.625	68.18 ± 1.8
*S.* Typhimurium ATCC14028	20–35	25	1.25	116.67 ± 20.4

^a^ O-antigen length of *S.* Typhimurium.

^b^ DOC, deoxycholate.

^c^ The average survival rate (means ± SD).

The length of LPS OAg chains is thought to determine complement resistance [12]. Thus, the complement sensitivities of the *wzz* mutants to WT were compared. ATCC14028 was discovered to be highly resistant to complement-mediated killing (survival rates of >100% after 1 h incubation). ST03 had about two-fold lower killing susceptibility than the WT, whereas ST04 shows a ranking-conscious complement resistance in between (Table 3).

The above findings show that LPS OAg length affected bacterial colonization, cell permeability, antibiotic resistance, and complement resistance in *S*. Typhimurium.

### The effect of OAg length on humoral immune responses in mice

**Figure 3:**
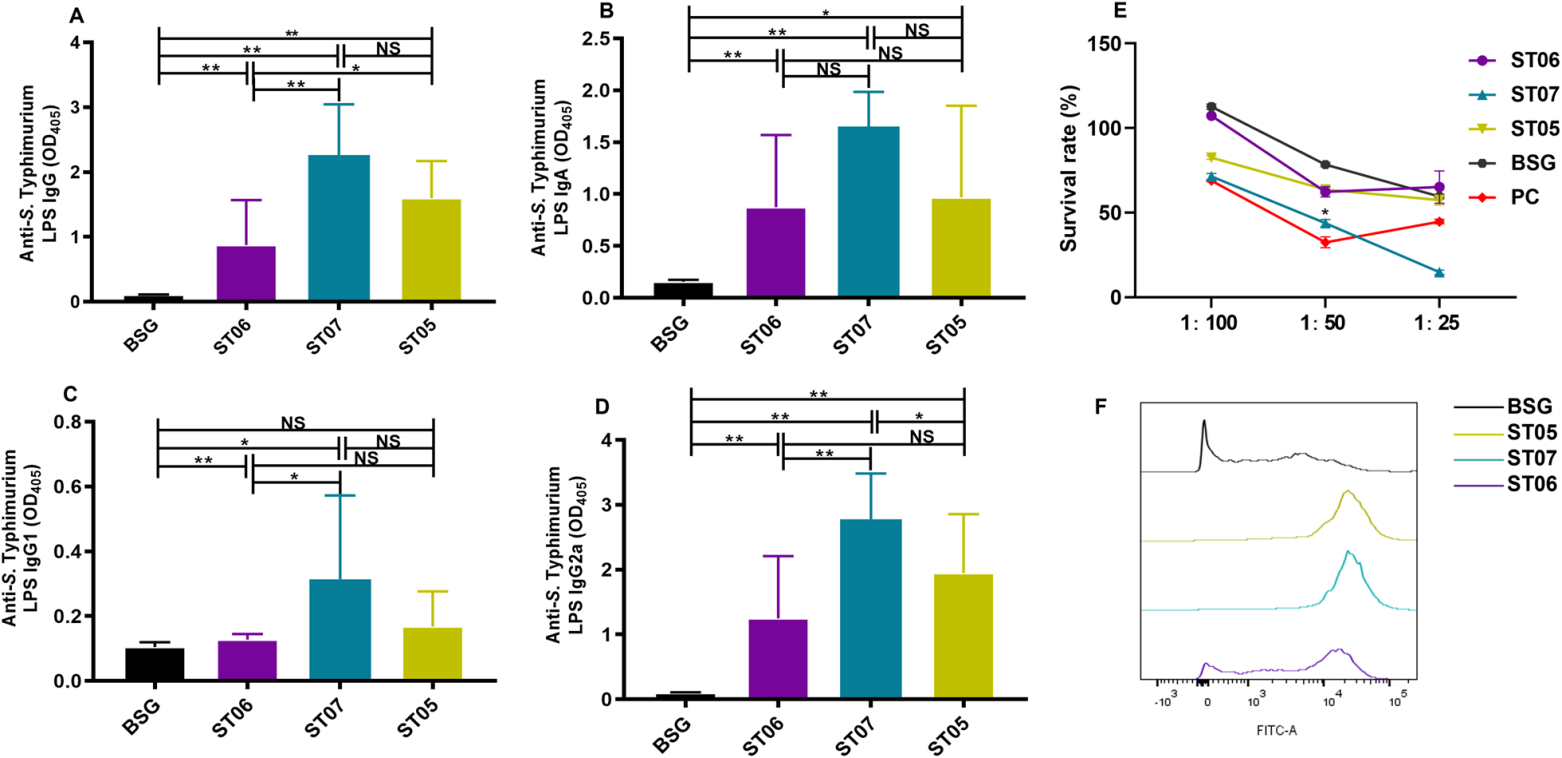
**Antibody immune responses induced by *S*. Typhimurium with variable OAg lengths in mice.** Serum IgG (**A**), IgA (**B**), IgG1 (**C**), and IgG2a (**D**) specific for *S*. Typhimurium LPS were measured by ELISA. Error bars represent the standard deviation of 5 replicate samples. *P < 0.05, **P < 0.01 assessed by two-way ANOVA. **E** Serum bactericidal assay. Serum collected from BSG, ST06, ST07, and ST05-immunized groups and commercial rabbit serum against *salmonella* O4 (PC) were reacted with wild-type ATCC14028 to determine the survival rate of *S*. Typhimurium. Error bars represent the standard deviation of 3 replicate samples. **F** Flow cytometry histograms of C3 complement deposition. Antibody deposition on *S*. Typhimurium in 10% pooled antiserum from each group of attenuated *S*. Typhimurium differing for OAg length immunized mice. Wild-type *S*. Typhimurium incubated with sera from the BSG-inoculated group was set as the negative control (black line), incubated with sera from the ST05 (ATCC14028Δ*crp*Δ*cya*)-inoculated group was set as the positive control (green-yellow line). The blue line indicates incubation with sera from ST06 (ATCC14028Δ*wzz*_ST_Δ*crp*Δ*cya*)-inoculated group, and the purple line indicates the incubation with sera from mice immunized with ST07 (ATCC14028Δ*wzz*_ST_::*wzz*_ECO2_Δ*crp*Δ*cya*).

The immune protective efficacy against *Salmonella* of the modified delivery vector was first evaluated in a murine model. After serum samples were collected from mice, *S*. Typhimurium LPS-specific IgG and IgA antibodies were evaluated using an ELISA on LPS-coated plates. Compared to the BSG control group, all strains generated more significant *S*. Typhimurium LPS-specific IgG (Figure 3A), with ST07 (ATCC14028Δ*wzz*_ST_::*wzz*_ECO2_Δ*crp*Δ*cya*), eliciting a significantly higher level than ST06 (ATCC14028Δ*wzz*_ST_Δ*crp*Δ*cya*) and a slight increase when compared to the ST05 (ATCC14028Δ*crp*Δ*cya*) vaccinated group. Similar trends were also observed in the IgA (Figure 3B), IgG1 (Figure 3C), and IgG2a (Figure 3D) level detections. Meanwhile, all the immunized groups induced a significantly higher level of IgG2a specific to the *S*. Typhimurium LPS compared to IgG1 was observed in the groups, indicating a predominantly Th1-type response.

The serum bactericidal activity was also detected to evaluate the humoral immune response. The results show that the percentage of the surviving bacteria increased as the serum concentration was diluted. After being incubated with serum at a dilution of 1:25, the ST07 group had the lowest survival rate, even lower than the positive control (rabbit serum against *Salmonella* O4) (*P* < 0.05). When incubated with serum at a dilution of 1:50, the ST07-immunized group had a significantly lower number of surviving bacteria than ST05, ST06 and BSG groups (*P* < 0.05). When incubated with serum at a dilution of 1:100, the ST07 group had a comparable survival rate to that of the ST06 group (*P* > 0.05) (Figure 3E). According to the results, the antibodies elicited in the ST07 group possess a powerful capacity for bacterial killing.

The antigen and antibody complex would deposit complement if the bacterial killing was antibody-dependent. Then the complement deposition efficiency was determined by flow cytometry. When incubated with sera from mice immunized with ST07 (ATCC14028Δ*crp*Δ*cya*Δ*wzz*_ST_::*wzz*_ECO2_) (blue line), the deposition of C3 on the surface of* S*. Typhimurium was higher than that collected from the ST06 (ATCC14028Δ*crp*Δ*cya*Δ*wzz*_ST_) (purple line) immunized group and even exceeded the positive control of the ST05 group (ATCC14028Δ*crp*Δ*cya*) (green-yellow line) (Figure 3F). The result shows that bacterial OAg length was related to antibody level and functional activity in bacterial killing, as well as complement deposition efficacy of sera.

### The protective efficacy in mice

To see whether the protective immunity was due to the OAg length-dependent serum activity, mice were challenged with 5 × 10^7^ CFU (~ 100 LD_50_) of *S*. Typhimurium 2 weeks after the booster. All the immunized groups conferred 100% protection, more significantly than the BSG-treated group (*P* < 0.05). This could be attributed to the reason that outer membrane immunogens other than modified LPS played functional roles in eliciting immune protection against *S*. Typhimurium. A comparable previous study showed that outer membrane vesicles (OMV) from gene deletion implicated in LPS synthesis also induced good protection against *S*. Typhimurium, which could be a reasonable explanation [28].

### The LPS-specific CD4^+^ T-cell immune response in mice

**Figure 4:**
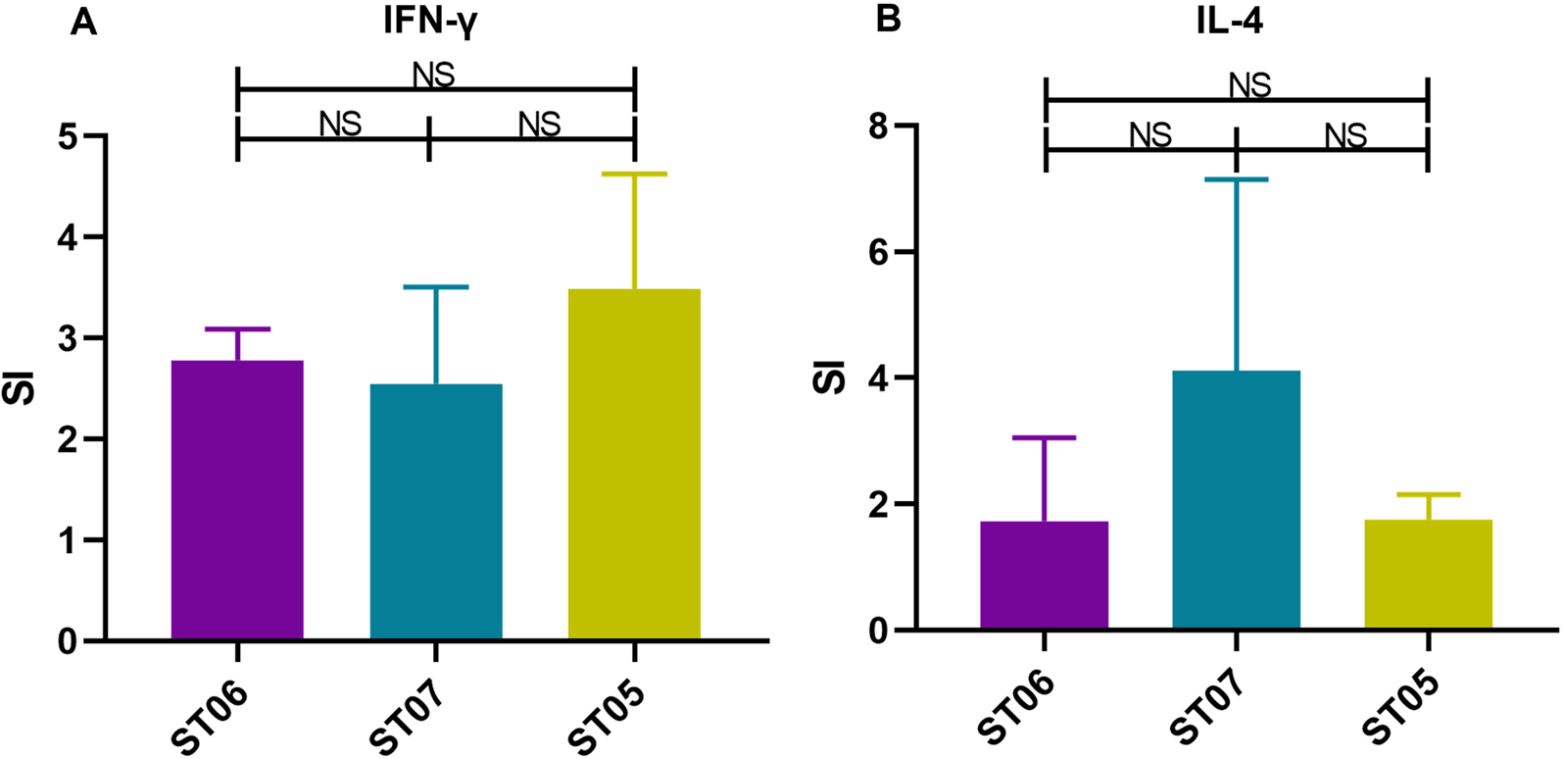
**Cytokine expression in CD4**^**+**^** T cells stimulated with *S*. Typhimurium LPS ex vivo.** After immunization in mice, the cytokine stimulation index (SI) was expressed relative to the percentage of IFN-γ (**A**) or IL-4 (**B**) cell subsets in the BSG-treated group. Data from two independent experiments were pooled and analyzed (*n* = 4) by t-test and two-way ANOVA. NS = not significant. Error bars represent the standard deviation of 4 replicate samples.

T-cell immunological responses across these strains were also detected after immunization. IFN-γ was selected to reflect a Th1 immune response, whereas IL-4 is a cytokine that indicates Th2 immunological responses. Flow cytometry tests were used to assess the LPS-specific CD4^+^ T-cell responses generated by the OAg length variation strains after stimulation of splenocytes in vitro with *S*. Typhimurium LPS. The result shows that the ST07 (Δ*crp*Δ*cya*Δ*wzz*_ST_::*wzz*_ECO2_) elicited a similar IFN-γ level to the ST05 (Δ*crp*Δ*cya*) and ST06 (Δ*crp*Δ*cya*Δ*wzz*_ST_) (Figure 4A), and a higher IL-4 level than that of ST06 and ST05 without a statistical difference (Figure 4B). The level of IL-4 cytokine was higher than that of IFN-γ, indicating that Th2-type cytokines play a dominant role in response to *S*. Typhimurium infection of the host immune system.

### Immune protection evaluation of recombinant O2-*Salmonella* in chickens

**Figure 5:**
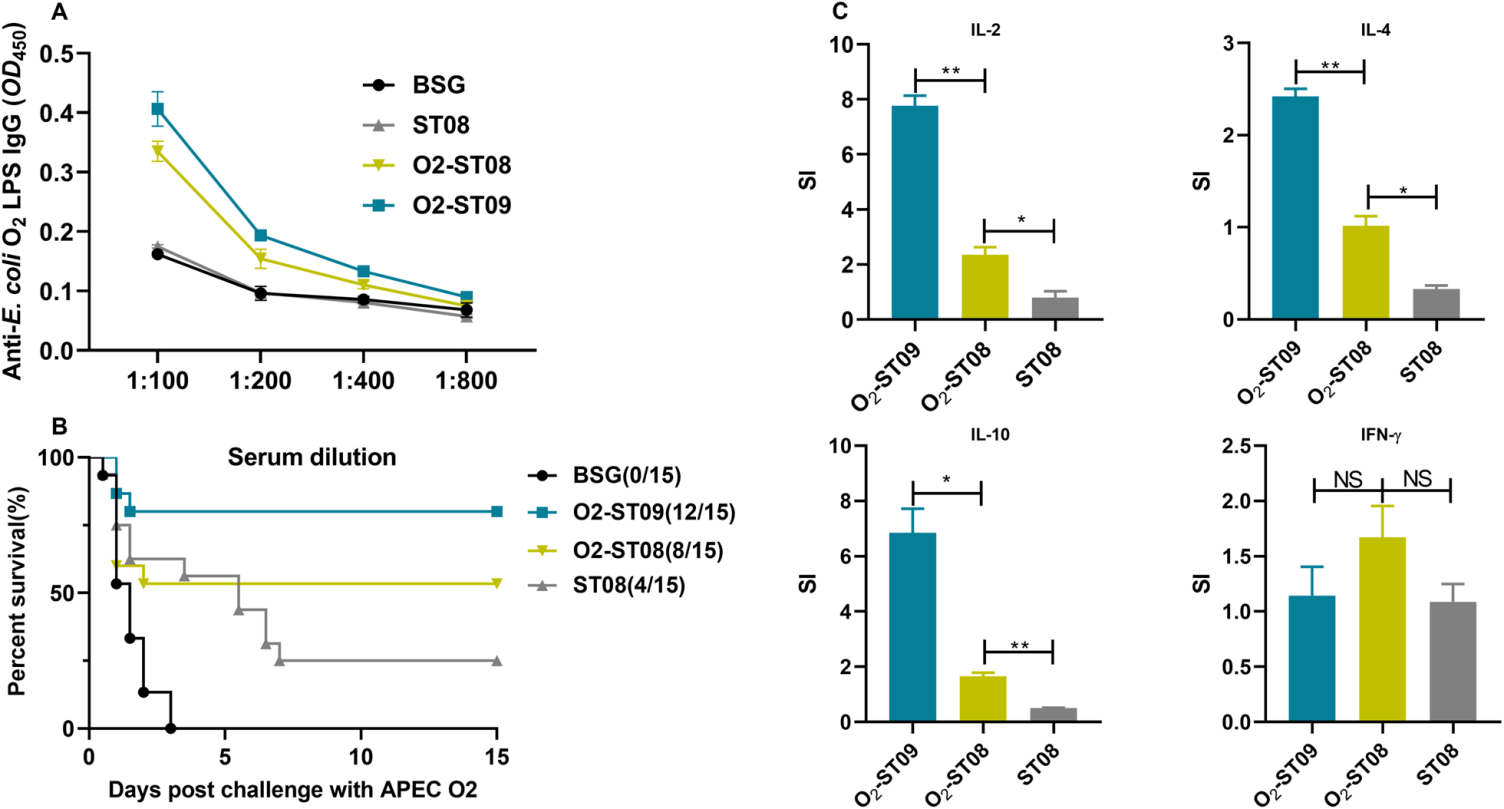
**Immune protective evaluation in birds.** **A** Antibody level detection, **B** Survival rate, **C** mRNA level of cytokines elicited in immunization group, the cytokine stimulation index (SI) was expressed relative to the percentage of IL-2, IL-4, IL-10 and IFN-γ mRNA level in the BSG-treated group. Data were analyzed (*n* = 5) by t-test. *P < 0.05, **P < 0.01, NS = not significant. Error bars represent the standard deviation of 5 replicate samples.

To determine whether Δ*wzz*_ST_::*wzz*_ECO2_ mutant has superior immunization potential in delivering heterologous *E. coli* O2 OAg polysaccharide, the APEC O2 O-polysaccharide gene cluster carried by plasmid pSL2162 was transformed into ST09 (ATCC14028 Δ*wzz*_ST_::*wzz*_ECO2_Δ*crp*Δ*cya*Δ*rfbP*Δ*asd*), resulting in the recombinant strain O2-ST09. The protective efficacy was evaluated in chickens and compared to that of O2-ST08 (ATCC14028Δ*asd*Δ*crp*Δ*cya*Δ*rfbP*). Since humoral immunity is an essential index in evaluating the protection of the APEC vaccine, serum IgY specific to APEC O2 LPS was detected by ELISA seven days after secondary immunization. The serum antibody level of IgG isolated from the O2-ST09 immunized group was 1:400, modestly higher than that elicited in the O2-ST08 immunized group and significantly higher than the BSG and ST08 control groups (Figure 5A). The result indicates that specific immune response against O2 LPS was elicited after immunization with strain O2-ST09, which possess 13–21 RU (Figure 5A).

After the challenge with APEC O2 wild-type strain DE17, the mortality was recorded daily for 15 days. The survival rates of chickens immunized with O2-ST09, O2-ST08, and ST08, were 80.00%, 53.33%, and 26.67%, respectively (Figure 5B). Furthermore, the O2-ST09 immunized group maintains an average weight gain with the O2-ST08 immunized group and the control group (Table 4).

**Table 4:** **Weight gain of Xiaoshan chickens from 1 to 4 weeks**

Age	Groups (average ± SD) (g)			
	BSG	ST08	O_2_-ST08	O_2_-ST09
1^st^ week	48.66 ± 7.79	48.66 ± 7.79	48.66 ± 7.79	48.66 ± 7.79
2^nd^ week	98.59 ± 17.63	113.85 ± 11.78	116.25 ± 11.47	111.51 ± 9.58
3^rd^ week	174.28 ± 29.06	192.3 ± 23.36	197.39 ± 18.69	187.7 ± 19.91
4^th^ week	/^a^	195.41 ± 39.76	224.48 ± 25.97	210.31 ± 26.33

^a^ BSG control group, the body weight was not calculated since only one chicken survived until weighing.

The results reveal that *E. coli* O2 OAg synthesized in *S*. Typhimurium with 13–21 RU could help trigger better immune protection against *E. coli* O2, which provides a better way to prevent *E. coli* O2 infection.

### mRNA level of cytokines in chickens

Data from qPCR tests show that the O2-ST09 immunized group had higher mRNA levels of IL-4, IL-2 and IL-10 than the O2-ST08 immunized group and the BSG control group (*P* < 0.01). There was no significant difference in IFN-γ eliciting (Figure 5C). The increase of IL-2 and IL-4 suggesting that the activation of CD4^+ ^T cell and the differentiation of Th2 cells were promoted. The enhanced secretion of IL-10 may be caused by the promotion of Th2 differentiation.

### Histopathological analysis and immunohistochemical staining of the liver and spleen from chickens

**Figure 6:**
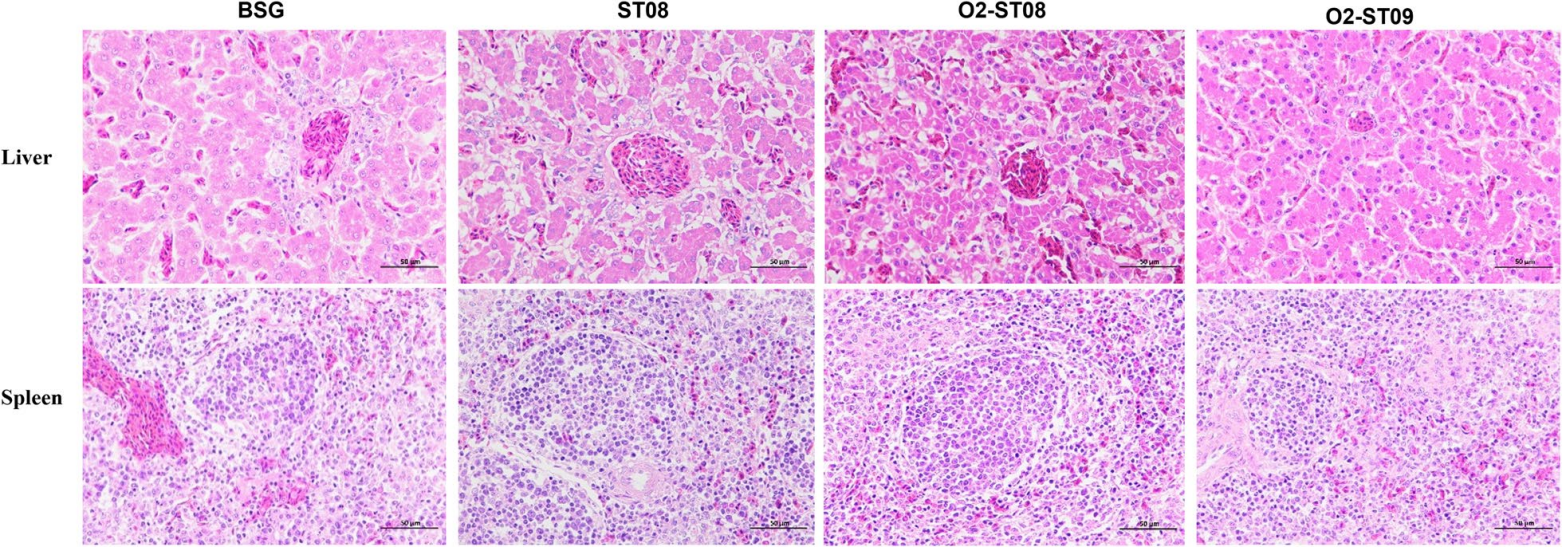
**Representative images of histopathological changes in birds.** Sections were stained with HE for pathological examination. Gross lesions and immune infiltrate were visually assessed. Scales bars = 50 μm.

Histopathological lesions in chickens were compared in the immunized groups BSG, ST08, O2-ST08 and O2-ST09 after the challenge with the wild-type strain DE17. The BSG control group had liver congestion, inflammatory cell infiltration, spleen congestion, and immune cell disorders, as illustrated in Figure 6. Chickens vaccinated with ST08 showed signs of degeneration and congestion. The O2-ST08 group had a disorganized liver with congestion. The O2-ST09 group showed slight degeneration and congestion of liver cells. No remarkable clinical signs of illness were observed in the spleens of groups vaccinated with O2-ST09, O2-ST08 and ST08. Furthermore, the BSG and ST08 control groups had clinically typical pericarditis and spleen enlargement, but the O2-ST08 and O2-ST09 immunized groups, particularly the O2-ST09 immunized group, had relatively minor clinical lesions (Additional file 1).

**Figure 7:**
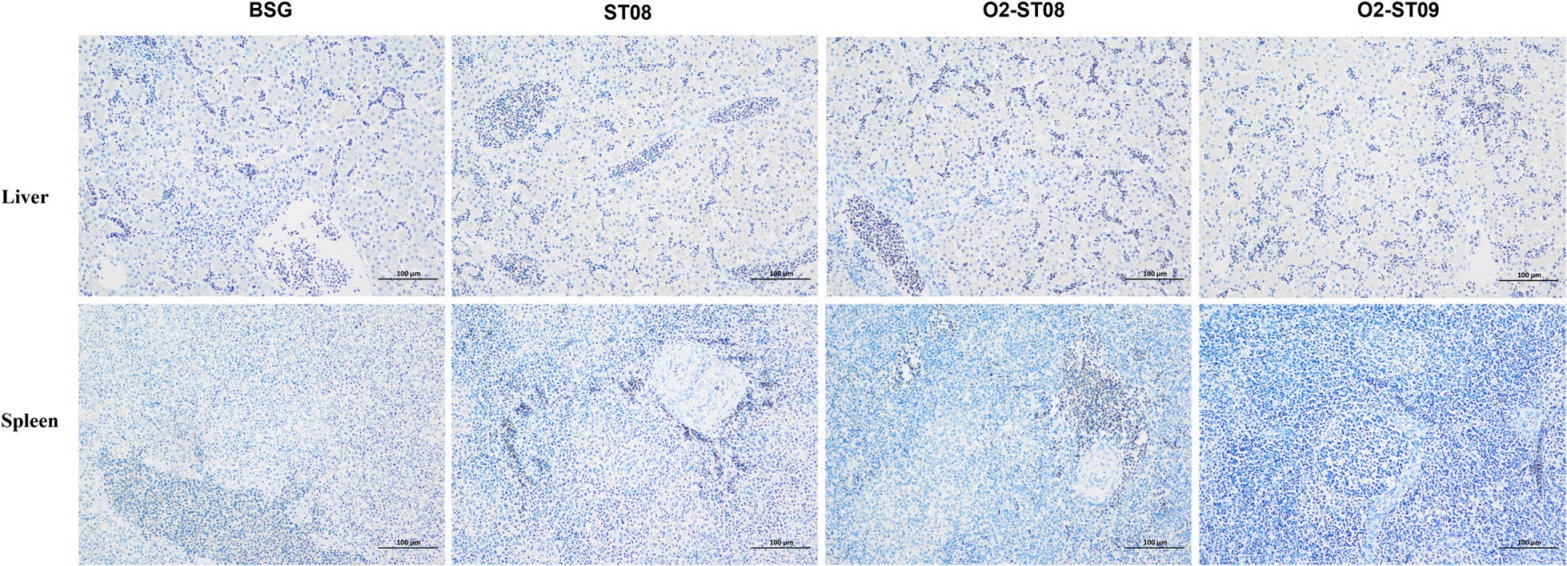
**Representative images of immunohistochemical staining of *E. coli* O2 after challenge in birds.** Immunohistochemical staining were performed on paraffin-embedded section of chicken liver and spleen tissues. The hematoxylin-stained nucleus are blue, DAB positive expression is brownish yellow. Scales bars = 100 μm.

We conducted immunohistochemistry using the rabbit anti-*E. coli* O2 antibody to confirm whether the immunization can confer protection against the *E. coli* O2 challenge*.* The brownish-yellow area represents the challenge strains that were not neutralized by antibodies induced by immunization. No significant differences in the brownish-yellow area were observed in liver cells among the investigated groups (Figure 7). In the spleen, the brownish-yellow area was detected in the BSG, ST08 and O2-ST08 immunized groups; no obvious reaction area was found in the splenic cells of the O2-ST09 immunized group (Figure 7).

## Discussion

LPS is a common component of cell envelopes, accounting for approximately 70% of gram-negative bacterial outer membranes [29]. The length of the OAg varies the most, ranging from a single repeat unit of 3 to 4 sugars to more than one hundred sugar units, resulting in an efficient extracellular barrier [30]. A previous study has shown that the OAg length influences the magnitude and quality of the immune response elicited by OAg-conjugate vaccines [31]. Because different *wzz* genes produce different OAg sizes, the *wzz* can be used to manipulate the polysaccharide length, altering the immunogenicity of the strains [32].

In this study, we assessed the effect of OAg length on *S.* Typhimurium immunogenicity by expressing the *wzz* gene from *E. coli.* All these bacteria depend on the Wzx/Wzy pathway to synthesize LPS. In the following experiment, we used a single variable factor by changing the OAg length determinator *wzz* without considering the *fepE*-encoded VL OAg chain length (HMW polysaccharide), generating a series of mutant strains of Δ*wzz*_ST_, Δ*wzz*_ST_::*wzz*_ECO2_. The immune response detection shows that the mutant* S*. Typhimurium ST07 (Δ*crp*Δ*cya*Δ*wzz*_ST_::*wzz*_ECO2_) stimulated a slightly higher IgG level in mice than in the other strains that were tested, indicating that ST07 triggered a strong humoral immune response to *S*. Typhimurium LPS. The recombinant strain O2-ST09 (Δ*asd*Δ*crp*Δ*cya*Δ*rfbP*Δ*wzz*_ST_::*wzz*_ECO2_) with medium OAg length induced more significant functional IgG antibodies and elicited more robust immune protection than O2-ST08 (Δ*asd*Δ*crp*Δ*cya*Δ*rfbP*Δ*wzz*_ST_) with long OAg length. Early research found that the medium-sized oligosaccharides end-linked to carriers had high immunogenicity efficacy in the protection evaluation of licensed *Haemophilus influenzae* type b and *Neisseria meningitidis* glycoconjugates [33]. Additionally, short-chain Vi-polysaccharide induced a more prolonged proliferation of Vi-specific B cells in the spleen than the long-chain Vi-CRM197 conjugate [34]. Our results which were consistent with these findings, show that the *Salmonella* delivery vector with a medium OAg length has an advantage in eliciting a polysaccharide-specific immune response. Conflicting results, however, have been reported, such as large-sized OAg glycoconjugates (HMW-TT) eliciting a much lower IgG titer than smaller-sized OAg glycoconjugates (LMW-TT) and low-molecular-mass OAg fragment eliciting notably higher antibody levels in mice than full-length OAg [35]. Two hypotheses are being considered, with one explanation being the various vaccine forms. Previous research in *Shigella*, *Francisella tularensis*, or *S*. Typhimurium usually refers to the protein-conjugated polysaccharide vaccine, from which LPS was extracted and conjugated to a carrier protein, such as TT, whereas glycan chain length is affected by various factors and conditions used during glycoconjugate vaccine production [33]. In contrast, the polysaccharide reported here was biosynthesized in a live *S*. Typhimurium strain, and the extraction methods had no effect on LPS length, affinity, or avidity. Another possibility is that the chain length was defined inconsistently; if the VL OAg length is responsible for HMW and the L OAg length is responsible for LMW, the results obtained here were reached by modifying LMW while keeping HMW rather than directly comparing with HMW and LMW. So, we assume that the classification method is another factor affecting the conclusion reached.

The anti-LPS antibody response profile was dominated by a Th2 immunological response. Exactly, live attenuated *Salmonella* has the advantage of effectively presenting antigens to dendritic cells and is favored for their ability to trigger a robust humoral immune response. Therefore, the antigen-specific CD4^+ ^T cell immune response was also detected. As expected, ST07 induced a similar IFN-γ and IL-4 level to ST05 in mice, and the CD4^+^ T cell immune response to *S*. Typhimurium LPS was not decreased after the mutation of *wzz*_ST_::*wzz*_ECO2_. Furthermore, compared to O2-ST08, the O2-ST09 immunized group even elicited a higher mRNA level of IL-2, IL-10 and IL-4 in chickens, each of which is necessary for the proliferation of activated T cells, the regulation of Treg cells and the promotion of Th2 cells, respectively. This suggests that the intermediate OAg length plays a role in the induction of an optimal Th2-biased immune response and is directly related to the immunogenicity of *S*. Typhimurium, particularly for the delivered polysaccharide.

Previous studies have shown that LPS was chosen as a gateway for antibacterial proteins to disrupt bacterial OM and perform antibacterial actions because of its conserved structure and influence on outer membrane protein behavior and presentation because most antibiotics are currently directed at intracellular processes and must be capable of penetrating the bacterial cell envelope [36]. Moreover, altering these molecules has a significant impact on bacterial sensitivity to various antibiotics as well as drug resistance [37]. Polymyxin B and DOC, for example, are LPS chemotype sensitive [38, 39]. In addition, LPS mutations are responsible for quinolone (e.g., Norfloxacin) and beta-lactam (e.g., Ampicillin, Penicillin) resistance [40]. Thus, we postulated that the antibiotic receptor would be exposed if OAg length shortens, allowing for a lower minimum inhibitory dose of polymyxin B for *S*. Typhimurium and increasing the sensitivity to NOR, ampicillin, and penicillin. Moreover, the changing of OAg length could alter the OM landscape by influencing inner membrane protein responses for cell envelope stability and colicin secretion, thus influencing bacterial resistance to DOC and tetracycline [41].

Cell membrane permeabilization is one of the best-known mechanisms of antibiotic activity [42]. LPS covers a large portion of the cell surface, creating a permeability barrier that keeps harmful chemicals such as antibiotics and bile salts from entering [43]. We found that decreasing the length of the OAg molecule reduced cell membrane permeability. This change in permeability may aid *Salmonella* adaptation to specific environmental conditions, which would explain why the bacteria are sensitive to polymyxin B and DOC. It is not known how exactly OAg length reduces membrane permeability. The enterobacterial common antigen (ECA) is a surface antigen related to OAg that plays a role in membrane maintenance [44]. It remains to be seen whether varying the length of the OAg influences ECA and, subsequently, the permeability barrier of the outer membrane (OM).

In addition to chemical warfare, bacteriophage infections and the immune system all rely on the cell membrane as the first line of defense against germs [45]. As a result, we identified the effect of strains with varying OAg lengths on complement-mediated killing and complement resistance deposition. Our data demonstrate that the mutant strain with medium OAg length produced more antibodies involved in the complement-mediated bactericidal activity and complement deposition than strains with either long or short chains. This is partly because strains with long chains produce blocking antibodies to impair complement-mediated bacterial killing, while strains with short-chains create fewer epitopes and blocking antibodies. The results support an earlier finding that *S*. Typhimurium strains with shorter OAg side chains are more susceptible to serum [21, 46]. In addition, antibodies against long OAg inhibit serum killing [47]. Even the previous study suggested that *E. coli* O157 strains with medium or long-length OAg chains may be more resistant to serum complement than those with short chains [13]. It is suspected that the phenomenon is because LPS OAg, composed of a variable number of repeat units, containing more than 100 RUs in a single cell, provides a formidable barrier to limit the access of the antibody to the bacterial surface. Changing the length of OAg would modify the chemical and physical structure of LPS OAg and the presentation of specific epitopes within proteinaceous surface antigens [48]. The protective quality of the Δ*wzz*_ST_::*wzz*_ECO2_ in complement resistance indicates that the type of OAg repeat presented plays a role as well, confirming that the length of the OAg side chain regulates bacterial resistance to the serum complement system [49]. More research is required to understand how the LPS-specific antibodies interact with the surface of bacteria.

In summary, we provide evidence that the LPS OAg length regulator *wzz* significantly affects *S*. Typhimurium. Furthermore, the recombinant strain O2-ST09 derived from Δ*wzz*_ST_::*wzz*_ECO2_ contributed to the protective efficacy in chickens. These findings expand our knowledge of the influence of OAg determinant Wzz on *Salmonella* and the polysaccharide delivered.

## Supplementary Information

**Additional file 1. Representative images of clinical and autopsy changes.** The severity of heart and spleen lesions were assessed to evaluate the immune protective efficacy against APEC O2 challenge. Gross lesions were evaluated visually.

## Data Availability

All data generated or analyzed during this study are included in this published article.
