# Supplementary figures and images for: The effect of O-antigen length determinant wzz on the immunogenicity of Salmonella Typhimurium for Escherichia coli O2 O-polysaccharides delivery
Source: Vet Res. 2023 Feb 27;54:15. doi: 10.1186/s13567-023-01142-4 (PMC9969949; doi:10.1186/s13567-023-01142-4)

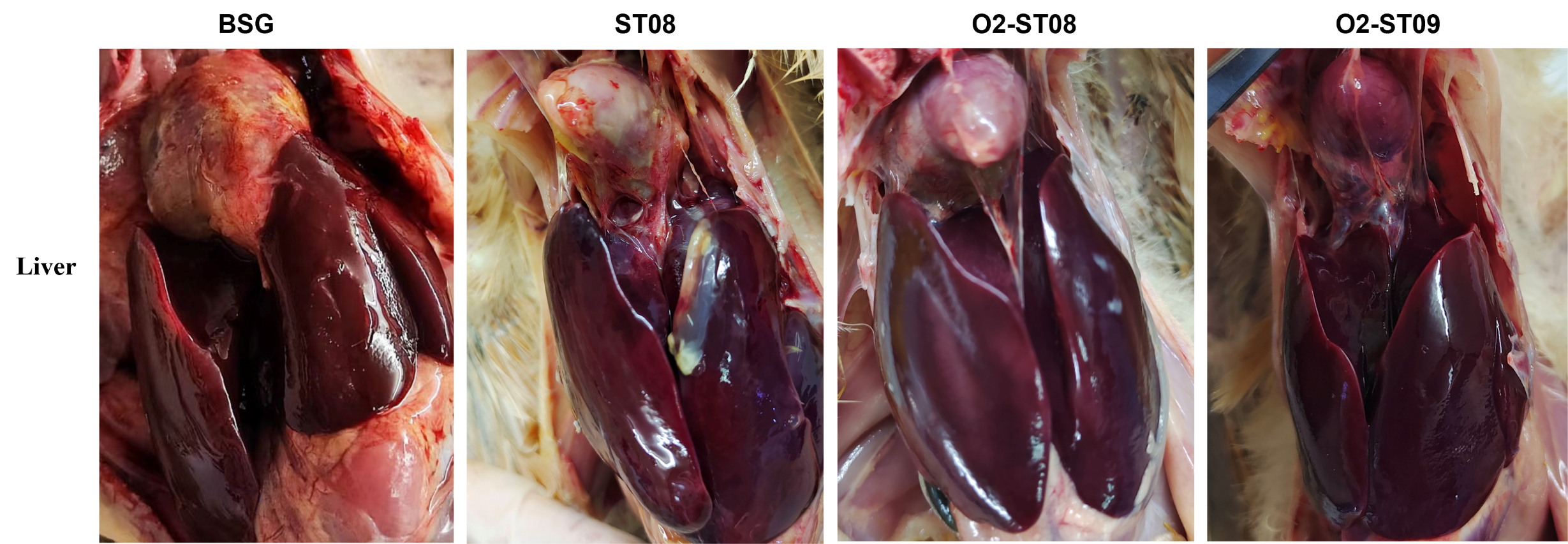

Supplement: Supplementary file 1 — Additional file 1. Representative images of clinical and autopsy changes. The severity of heart and spleen lesions were assessed to evaluate the immune protective efficacy against APEC O2 challenge. Gross lesions were evaluated visually. [file 13567_2023_1142_MOESM1_ESM.tif]
